# Descriptive Analysis of Pre-existing Data on Eosinophilic Esophagitis and Associated Morbidities in Cleveland Clinic Abu Dhabi, United Arab Emirates

**DOI:** 10.7759/cureus.51493

**Published:** 2024-01-01

**Authors:** Mohamad Abuzakouk, Osama Yousef, Monica Fahmy, Zahir Chouikrat

**Affiliations:** 1 Allergy & Immunology Department, Respiratory Institute, Cleveland Clinic Abu Dhabi, Abu Dhabi, ARE; 2 Digestive Disease Institute, Cleveland Clinic Abu Dhabi, Abu Dhabi, ARE; 3 Medical Department, Sanofi, Dubai, ARE

**Keywords:** co-morbidities, eosinophilic gastrointestinal disorders, real-word evidence, united arab emirates, eosinophilic gastritis/gastroenteritis, eosinophilic esophagitis

## Abstract

Background: Data on eosinophilic gastrointestinal disorders (EGIDs) are limited in the United Arab Emirates.

Objective: This study aimed to describe the distribution of eosinophilic gastritis/gastroenteritis (EoG/EGE) and eosinophilic esophagitis (EoE) among patients whose data are recorded on the Cleveland Clinic Abu Dhabi (CCAD) systems.

Methods: Anonymized aggregate data were obtained from the Clinerion Patient Network Explorer (PNEx^®^) network installed at the CCAD on patients diagnosed with EoG/EGE (n=17, of whom 11 (64.7%) were females) or with EoE (n=118, of whom 35 (29.7%) were females) between 2015 and 2022; 63 (53.4%) were diagnosed between 2020 and 2022.

Results: The vast majority of EoE patients visited the gastroenterology clinic (111, 94.1%), followed by the primary care (70, 59.3%), the emergency medicine (60, 50.8%), and the immunology (59, 50%) units. A total of 84 patients with EoE also presented with another type-2 inflammatory condition including allergic rhinitis, food allergy, and atopic dermatitis. Proton pump inhibitors and corticosteroids were widely prescribed, and four patients with EoE have been commenced on biologics since 2020. However, heterogeneity of medication use was identified, warranting local clinical practice guidelines.

Conclusion: A referral network could be established with local and regional reach, which relies on a multidisciplinary approach for the management of EoE and associated type 2 co-morbidities. More studies are needed to better depict the clinical picture of patients with EGIDs in general and EoE, in particular, in terms of diagnosis, pharmacological treatment, non-medical interventions, disease course, and patient-reported outcomes.

## Introduction

Eosinophilic esophagitis (EoE; International Classification of Diseases, 10th Revision (ICD-10), code K20.0) is a chronic inflammatory disorder increasingly recognized as a cause of feeding problems in children and dysphagia and food impaction in adults [1]. EoE is clinically distinct from gastroesophageal reflux disorder and is characterized by signs and symptoms of eosinophilic infiltration, including narrowing and scarring of the esophagus and esophageal dysfunction, particularly dysphagia, food impaction, and chest pain [2-6]. There are three criteria required for EoE diagnosis: symptoms of esophageal dysfunction, eosinophilic inflammation localized to the esophagus with ≥15 eosinophils per high-power field in esophageal mucosal biopsy specimens, and exclusion of other causes of esophageal eosinophilia [7], while the trial of proton-pump inhibitors is no longer required in the diagnostic guidelines [8].

Early exposure (or lack of exposure) to environmental factors, lack of breastfeeding during infancy, excessive antibiotic exposure, and male gender might predispose individuals to an EoE onset [9-13]. EoE is, at least partially, mediated by downstream effects of interleukin (IL)-4, IL-5, and IL-13, which trigger a Th2-mediated inflammatory reaction [14-16]. The key and central type 2 inflammatory cytokines IL-4 and IL-13 promote infiltration of eosinophils into the esophageal mucosa and contribute to esophageal remodeling, including epithelial barrier dysfunction and hyperplasia, fibrosis, and smooth muscle hypercontractility [17,18].

The prevalence of EoE in Western populations was estimated at one to five per 10,000 persons [13,19]. Additionally, it has been trending upward in Asian population [20]. In the Middle East, the prevalence of EoE is not well-established. One decade ago, EoE was found in 45 patients over seven years at King Fahad Medical City [21]; in 15 patients (all males) diagnosed over three years (up to 2011) at the National Guard Hospital in Jeddah, Saudi Arabia [22]; and in 0.85% of children and adolescents in Western Saudi Arabia in 2012 [23]. In Jordan, at King Abdullah University Hospital, between January 2015 and January 2020, 21 patients were diagnosed with EoE (20 of whom were male children), making up to 3% of endoscopy-assessed cases [24]. Additionally, EoE prevalence was estimated at 2.6% among adults with esophageal symptoms in Turkey [25]. The reportedly low prevalence of EoE in the Middle East might be due to differences in screening and diagnosis or to oversight of patients whose EoE responds to acid suppression [26].

Eosinophilic gastritis (EoG)/gastroenteritis (EGE) is another manifestation of eosinophilic gastrointestinal disorders (EGIDs) [27], with a slight female and Caucasian race predilection [28,29]. EoG/EGE is diagnosed by morphological observation of biopsies, complementing a plethora of symptoms such as abdominal pain, nausea, vomiting, and diarrhea, as well as bowel obstruction and perforation in more severe cases [27]. EoG and EGE are EGIDs, which, like EoE, were considered independent of IgE antibodies, but are mediated by a Th2 cell inflammatory reaction and involve IL-2 and innate lymphoid cells in their pathogenesis [27,30-32].

Coexisting EoE was seen in 10-12% of patients with EoG/EGE in the United States [33], and these conditions might be associated with other atopic disorders. While corticosteroids are the mainstay of treatment of these diseases, biological agents might also offer therapeutic options, although no biologic has been approved for EoG/EGE and only one biologic (dupilumab) is approved for EoE [34].

Data at the Cleveland Clinic Abu Dhabi (CCAD) between 2015 and 2022 were analyzed to determine the prevalence of EoE and EoG/EGE in the adult population of Abu Dhabi in the United Arab Emirates and to depict patient profiles and associated co-morbidities in EoE and EoG/EGE.

## Materials and methods

Setting

Anonymized patient aggregate data were obtained from the Clinerion Patient Network Explorer (PNEx®) network installed at CCAD on adult patients diagnosed with EoG/EGE or with EoE between 2015 and 2022. CCAD is one of the best hospitals in Abu Dhabi with an institutional structure similar to that of the Cleveland Clinic Foundation (CCF) main campus in Cleveland, Ohio. CCAD is highly renowned for all the sub-specialties covered in the Gastroenterology & Hepatology Department, contributing to its position as one of the main referral centers in the region.

PNEx® generates data based on search results from a combination of criteria for diagnosis, medications, procedures, and laboratory results. No patient identifiers are reported in the PNEx®-generated data. The database holds electronic health records of over 300,000 patients.

The current study is a sub-analysis of a broader CCAD registry initiated for data collection on severe asthma and extended to type-2 inflammatory diseases, including eosinophilic esophagitis.

This study was approved by the Abu Dhabi Health Research and Technology Ethics Committee ADHRTC (DOH/CVDC/2023/465). No informed consent was required, as only anonymous data were compiled in an aggregate fashion and analyzed.

Analysis cohorts

Three cohorts were defined for analysis:

EoG/EGE cohort: 17 patients with EoG/EGE (ICD-10 code K52.81) between January 2016 and April 2022 (74 months),

EoE-A cohort: 118 patients with EoE (ICD-10 K20.0) between July 2015 and June 2022 (84 months), and

EoE-B cohort: 63 patients with EoE (ICD-10 K20.0) between January 2020 and June 2022 (29 months).

Patients diagnosed with EOE were divided into two groups, i.e., EOE-A and EOE-B, in an attempt to unveil any change in the disease management and treatment pattern with respect to the new guidelines and COVID-19 period.

Data extraction

In this study, anonymized records were obtained from electronic health records of patients with EoE and EoG/EGE between 2015 and 2022 from CCAD and reviewed in partnership with Clinerion in an aggregate fashion to determine the demographics of patients diagnosed with EoE and EoG/EGE in CCAD, their co-morbidities, and the treatment landscape between 2015 and 2022.

Statistical considerations

There was no formal sample size determination. The research scale was based on the total patient population treated for EoE and EoG/EGE at CCAD between July 2015 and August 2022 (total of 184 patients) in CCAD. Data were presented as frequencies and percentages for categorical variables. Numerical variables (age) were converted into categories and treated as categorical variables.

## Results

Incidence of EoG/EGE and EoE

Between February 2016 and April 2022, 17 cases of EoG/EGE were diagnosed, all as a primary diagnosis. In addition, 118 patients (EoE-A) were diagnosed with EoE between July 2015 and June 2022, all of whom as a primary diagnosis. Out of the 118 cases (0.076 per 100,000 population), 63 (EoE-B) were diagnosed within 29 months, extending from January 2020 to June 2022.

Patient presentation to CCAD 

The 17 patients with EoG/EGE presented to CCAD for a total of 35 times within the 74-month timeframe. EoE-A cohort patients presented to CCAD for a total of 295 times within the 84-month timeframe. However, within 29 months between 2020 and 2022, EoE-B cohort patients consulted at CCAD for a total of 140 times.

Among the 118 patients with EoE, 111 (94.1%) were managed by gastroenterologists (total of 424 visits) and 59 (50.0%) by allergy and immunology specialists (total of 329 visits; highest frequency of around 5.5 visits per patient). A substantial number of cases were also examined at the primary care level (70 patients, for a total of 185 visits), at the emergency medicine department (60 patients, for a total of 324 visits), and at the radiology department (66 patients, for a total of 129 visits). Additionally, 98 patients had laboratory analyses (a total of 455 laboratory tests), and 96 went through the perioperative department (a total of 152 visits).

Age and gender distribution of cases

**Figure 1:**
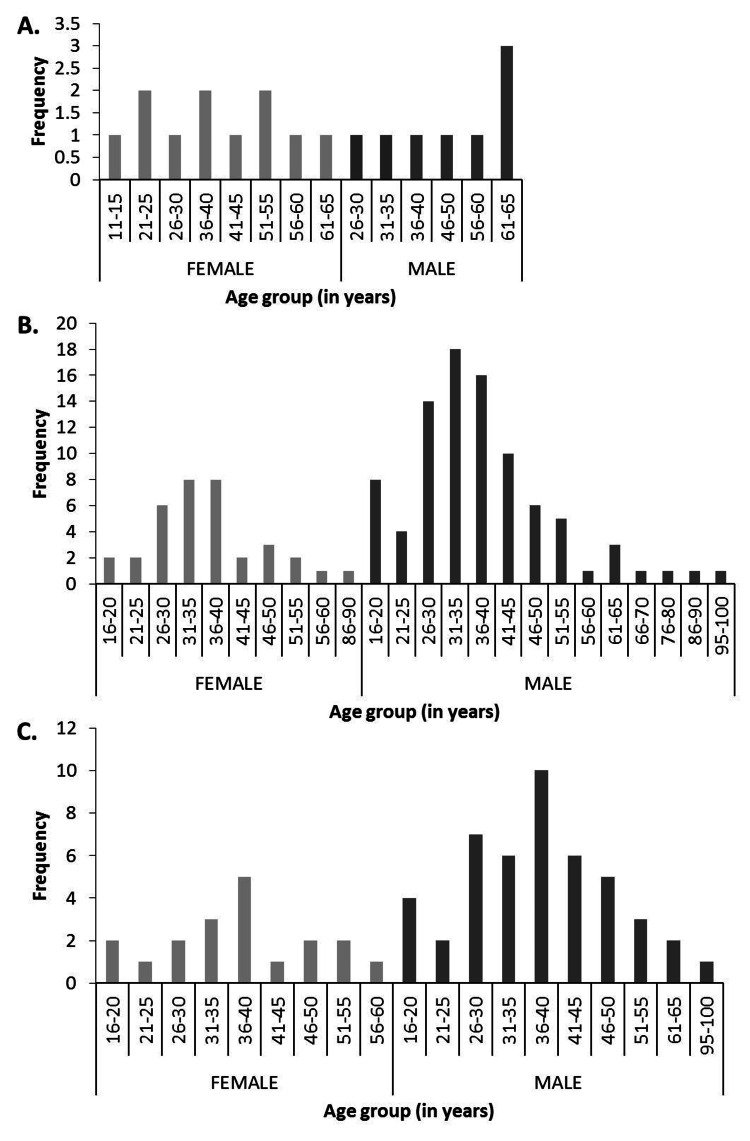
Age and gender distribution of patients with EoG/EGE and EoE A. Between 2015 and 2022, 17 patients were diagnosed with EoG/EGE at CCAD, of whom 11 (64.7%) were women. B. EoE-A cohort: Between 2015 and 2022, 118 patients were diagnosed with EoE at CCAD, of whom 35 (29.7%) were women. C. EoE-B cohort: Between 2020 and 2022, 63 patients were diagnosed with EoE at CCAD, of whom 19 (30.2%) were women. Some patients had visited the clinic more than once and had their data recorded more than once.

EoG/EGE did not have a sharp age or gender predilection, although the group with the highest frequency (n=3) was the 61-65-year-old male group (Figure 1A).

EoE had a male gender predilection, peaking between 31 and 40 years of age. Women between the ages of 26 and 40 years also had the highest incidence of EoE in their gender category, in EoE-A (Figure 1B) and EoE-B cohorts (Figure 1C).

Description of co-morbidities

**Figure 2:**
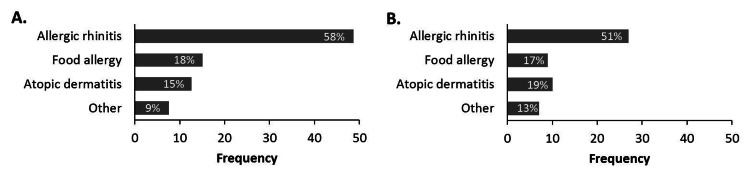
Type 2 co-morbidities reported for each of the EoE cohorts A. Proportion of co-morbidities reported for EoE-A cohort (n=84). B. Proportion of co-morbidities reported for EoE-B cohort (n=53).

Co-morbid conditions were analyzed for the two EoE patient groups. Figures 2A, 2B display specific type 2 inflammatory co-morbidities in association with EoE, showing that the pattern of allergic rhinitis, food allergy, atopic dermatitis, and other type 2 inflammatory conditions has not changed since 2015.

Treatment groups

In group EoE-A, out of 118 patients, prescription information was available for 100 (84.7%) patients. In group EoE-B, out of 63 patients, the prescription information was available for 46 (73.0%) patients. In group EoE-A, proton pump inhibitors have been prescribed 137 times since 2015, and in group EoE-B, these medications have been prescribed 52 times since 2020. Corticosteroids were also frequently prescribed with 86 prescriptions in the EoE-A group and 28 prescriptions in the EoE-B group. Omalizumab (n=1), benralizumab (n=2), and dupilumab (n=2) were prescribed to a total of five patients since 2015 and a total of four patients between 2020 and 2022.

A breakdown of medications given to more than 25% of patients can be found in Table 1. Mometasone has been prescribed 66 times since 2015, of which 11 times starting in 2020. On the other hand, prednisone was only prescribed starting in 2020. Pantoprazole was prescribed a total of 59 times, only eight of which after 2020.

**Table 1:** Medications prescribed to patients with EoE Data are displayed as the number of patients (number of prescriptions). EoE: eosinophilic esophagitis

	EoE-A (2015-2022)	EoE-B (2020-2022)
Medications of special interest		
Esomeprazole	72 (198)	31 (46)
Mometasone	28 (66)	9 (11)
Pantoprazole	22 (59)	6 (8)
Dexlansoprazole	21 (34)	11 (14)
Budesonide	15 (46)	6 (15)
Prednisone	---	5 (10)

**Figure 3:**
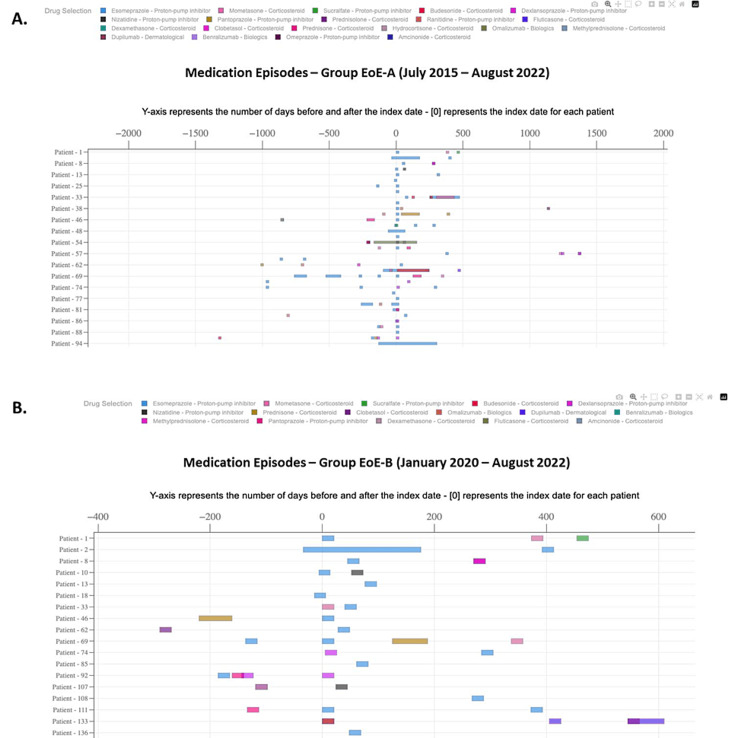
Medication patterns Group EoE-A, between July 2015 and August 2022. B. Group EoE-B, between January 2020 and August 2022. EoE: eosinophilic esophagitis.

A medication timeline has been created by looking at prescription data and measuring the distance between two consecutive prescription dates on each drug for each patient. The drug timeline was presented on the index date for each patient. The prescription data were limited to represent the information in the medication timeline. Figure 3 presents the medication patterns for the EoE cohorts. Data showed heterogeneity in treatment paradigm and varying duration of proton pump inhibitor and corticosteroid use. Within the scope of this study, no data were available on the therapeutic management of EoG.

## Discussion

EoE and EoG/EGE are conditions falling under the umbrella of EGIDs, and while EoE is better characterized and more recognized, EoG/EGE is a much rarer condition with poorly defined diagnostic criteria [27].

Globally, an overall estimate of the prevalence of EoE is 0.5-1 case per 1000 persons, and the condition has been on the rise with increasing awareness about its symptoms and clinical presentation [35]. However, little is known about EoE in the Arab world in general and in the United Arab Emirates in particular. The population of Abu Dhabi (home to the CCAD, a referral center for EGIDs) in 2022 was estimated at 1,540,000 [36]. In the present study, 118 patients were diagnosed with EoE in CCAD, making up 7.6 cases per 100,000 persons in Abu Dhabi.

Between 2016 and 2022, 17 patients were diagnosed with EoG/EGE at the CCAD, with a slight female predilection (11 women versus 6 men), but no further information was analyzed as part of the current study. In the absence of standardized diagnostic criteria for EoG/EGE, a diagnosis of exclusion is adopted [27, 37], leaving room for diagnostic errors.

Biopsies obtained from the esophagus of patients with EoE are characterized by at least 15 eosinophils per high-power field upon light microscopy observation [14], but this study did not examine pathological assessment of biopsies. This also limited the possibility of determining EoE severity among patients managed at the CCAD, knowing that the severity of EoE may inform the therapeutic approach [38]. In 2022, the Index of Severity for Eosinophilic Esophagitis (I-SEE) has been proposed as an accessible tool to evaluate EoE severity as part of routine clinical examinations [38].

The variety of specialists consulted by a large number of patients in the CCAD database attests to the attempt to diagnose and manage these conditions by a multidisciplinary team (MDT). In fact, recent literature promotes the management of EGIDs by an MDT, which includes a specialized dietician [39-41]. Diagnosis of EoE was mostly done at the gastroenterology clinic, without further information about sign and symptom presentation. It would, however, be interesting to report on the diagnostic context, as a recent study on a large Malaysian population reported a 2.6% rate of EOE diagnosis upon endoscopy for a variety of bowel-related ailments [42]. A large proportion of patients at the CCAD also presented with other type 2 comorbidities, in line with the literature [29] and reflected by the highest frequency of patient visits to the allergy and immunology department (with 59 patients visiting the clinics for a total of 329 times, a rate of 5.5 visits/patient). Additionally, 94% of the patients analyzed in this study had visited a gastroenterologist, emphasizing the incontestable importance of diagnosis at the gastroenterology clinic. The establishment of an MDT clinic concerned with EGIDs in the United Arab Emirates could serve as a local and regional referral center, similar to the Multidisciplinary Eosinophilic Esophagitis Clinic at the University of Virginia Digestive Health Center in the United States.

Corticosteroids and proton pump inhibitors were the most commonly prescribed medications, although one patient has been prescribed sequential medications of the same class. This might suggest that pharmacological treatment patterns are heterogeneous and seem to lack a protocol, underscoring the need for local guidelines. As reflected in the current analysis, corticosteroids remain the mainstay of EoE, although other non-steroidal pharmacological options are being assessed for use in EGIDs in general and in EoE [43] and EoG/EGE [27, 44] in particular. These non-steroidal medications include biological treatment with monoclonal antibodies (cendakimab, lirentelimab, benralizumab, mepolizumab) [45]. In the current analysis, five patients with EoE were prescribed a biological treatment between 2015 and 2022. Additionally, EoE in adults can benefit from non-pharmacological interventions, such as food elimination diets [46, 47] or the Elimination and Reintroduction protocol [48], as well as from endoscopic esophageal dilations for symptom relief [49,50].

Limitations

Given the data generation method, the patient journey could not be illustrated as timelines were not captured. Data on biopsy and endoscopic features, as well as on the severity of cases, could not be retrieved. Additionally, non-pharmacological or non-medical interventions such as dietary interventions or esophageal dilation were not reported.

## Conclusions

This analysis underscored the need for local guidelines to establish recommendations for diagnosing and managing EoE and EoG/EGE in the United Arab Emirates and the region. It also highlighted the importance of a multidisciplinary team approach in the management of type 2 eosinophilic conditions. A referral network could be established to serve patients in the Arab world, through centralized clinics specialized in eosinophilic esophagitis and associated morbidities. Additionally, more studies are warranted for a better understanding of the patient profile, treatment paradigm, and patient-reported outcomes, to feed into expanding registries that allow in-depth real-world evidence analyses.
